# Sexual function among controlled and uncontrolled hypertensive females receiving beta-blockers or ACEI/ARB and thiazides: a prospective randomized controlled study

**DOI:** 10.1038/s41598-026-40790-2

**Published:** 2026-03-17

**Authors:** Sameh Fayek GamalEl Din, Ehab Elyamani, Marina Talaat Bushra, Mahmoud Fawzy Ghaly, Mohamed Wael Ragab, Ahmed Fathy Aboseif

**Affiliations:** 1 Department of Andrology and STDs, Kasr Alainy Faculty of Medicine, Cairo University, Cairo, Egypt; 2 Department of Andrology and STDs Medicine, Beni-Suef University, Beni Suef, Egypt; 3 Department of Cardiology, Faculty of medicine, Beni-Suef University, Beni Suef, Egypt; 4 Egyptian Ministry of Population and Health, Cairo, Egypt

**Keywords:** hypertension, beta-blockers (BBs), angiotensin-converting enzyme inhibitors/ angiotensin receptor blockers (ACEIs/ARBs), thiazides, estradiol, total testosterone, free testosterone, female sexual function, Cardiology, Diseases, Drug discovery, Medical research

## Abstract

**Background:**

Female sexual dysfunction (FSD) among females with hypertension (HTN) is frequently overlooked, with a reported prevalence of 42.1%.

**Objectives:**

We aimed to determine the impact of beta-blockers (BBs), angiotensin-converting enzyme inhibitors/angiotensin receptor blockers (ACEIs/ARBs), and thiazides on sexual function in hypertensive females.

**Methods:**

A prospective randomized controlled trial enrolled 125 female participants. Group (1) included 25 normotensive females serving as the controls. Groups (2) and (3) consisted of 50 controlled and uncontrolled hypertensive patients who received BBs, respectively. Groups (4) and (5) consisted of 50 patients with controlled and uncontrolled HTN who received ACIs/ARBs, respectively. Each group consisted of patients who received one tablet daily of ramipril 2.5 mg for one month, while the other half received one tablet daily of valsartan (VAL) 80 mg for the same duration. After one month, the subjects were transitioned to a daily regimen of one tablet of ramipril 2.5 mg combined with hydrochlorothiazide 12.5 mg, as well as one tablet of VAL 80 mg with hydrochlorothiazide 12.5 mg for two months, respectively.

**Results:**

Controlled and uncontrolled hypertensive patients receiving ACEIs/ARBs, as well as controlled hypertensive patients receiving BBs, demonstrated a significant decrease in serum total testosterone and free testosterone levels, accompanied by a significant increase in estradiol after 3 months. Furthermore, controlled and uncontrolled hypertensive patients receiving ACEI/ARBs showed significant increases in all female sexual function (FSF) domains and total FSF scores after 3 months. Consistently, controlled hypertensive patients receiving BBs showed significant improvements across all domains of the validated Arabic version of the female sexual function index (ArFSFI) and the total score, comparable to the ACEI/ARB groups, except for pain. Conversely, uncontrolled hypertensive patients receiving BBs demonstrated significant increases in scores for desire and arousal and orgasm and satisfaction after 3 months. After three months, there was a significant reduction in the GAD-7 scores among all hypertensive patients.

**Conclusion:**

ACEIs/ARBs demonstrated a favorable effect on FSF. Future large-scale cohort studies are warranted to validate these findings as this study was a single center and of small sample size.

**Supplementary Information:**

The online version contains supplementary material available at 10.1038/s41598-026-40790-2.

## Introduction

Healthy sexual function significantly influences women’s quality of life^1^. Female sexual physiology results from a complex interplay of intrapsychic, relational, social, and neurovascular factors^2^. Following the onset of sexual stimulation, vasocongestion of clitoral tissue during arousal is mediated by nitric oxide (NO)^3^ and vasoactive intestinal peptide (VIP)^4^. Female sexual behavior and function are controlled by sex steroids and other hormones^5^. Estrogens are essentially synthesized from androgens, with estrone (E1) and estradiol (E2) are synthesized by the aromatization of androstenedione and testosterone (T), respectively^6^. Furthermore, adequate concentrations of free testosterone^7^ are central for NO to initiate vasocongestion in response to sexual stimulation. Vascular smooth muscle in the vagina is innervated by acetylcholinergic nerve fibers, leading to vaginal engorgement during arousal and subsequent lubrication^8^. It should be mentioned that the highest concentrations of E2 are detected in the hypothalamus and preoptic areas^9^ with subsequent impactful influence on sexual behavior^5^. Female sexual dysfunction (FSD) is characterized by orgasmic impairment, vaginal lubrication failure, vaginismus, and loss of libido^10,11^. FSD diagnosis necessitates the presence of distress that exceeds the observed symptoms or signs^12^. Despite a reported prevalence of 42.1%, FSD among hypertensive females is often overlooked as a clinical issue^13,14^. This elevated prevalence may be attributed to multiple mechanisms: hypertension (HTN) reduces vasodilation via increased arterial stiffness and impairs NO bioavailability through endothelial dysfunction, subsequently reducing vasodilatation and lubrication^15^. HTN also disrupts the central modulation of sexual behavior and impairs autonomic nervous system function^15^.

Oxidation and inflammation associated with HTN increase female genital tissue fibrosis, leading to subsequent damage of the endothelial cells^16^ that highlights the critical association between the inflammatory biomarkers and HTN^17^. Furthermore, HTN may cause anxiety and depression in certain females^13^. The primary applications of betablockers (BBs) include the treatment of HTN and angina pectoris, in addition to anxiety, tremors, and migraines^18^. Antihypertensives, such as BBs, are associated with sexual dysfunction, which can impact quality of life, overall well-being, and health^18^. In contrast, a study by Kumar et al. (2021) demonstrated a favorable impact of nebivolol compared to bisoprolol on the sexual function of hypertensive females^19^. Similarly, valsartan (VAL) has been demonstrated to improve sexual function in hypertensive females^20^. Nevertheless, the precise mechanism underlying the increased prevalence of FSD in hypertensive women has not been elucidated^1^. Consequently, the assessment of FSD should be incorporated into the clinical management protocols for women with hypertension^14^. We aimed to determine the impact of antihypertensives, including BBs or Angiotensin-Converting Enzyme Inhibitors (ACEI)/Angiotensin Receptor Blockers (ARBs) and thiazides, on sexual function in females with controlled and uncontrolled hypertension. Additionally, the study aimed to investigate the association between HTN and anxiety and depression in these patients, as well as the impact of these medications on the reproductive hormones in premenopausal patients.

## Patients and Methods

**Fig. 1:**
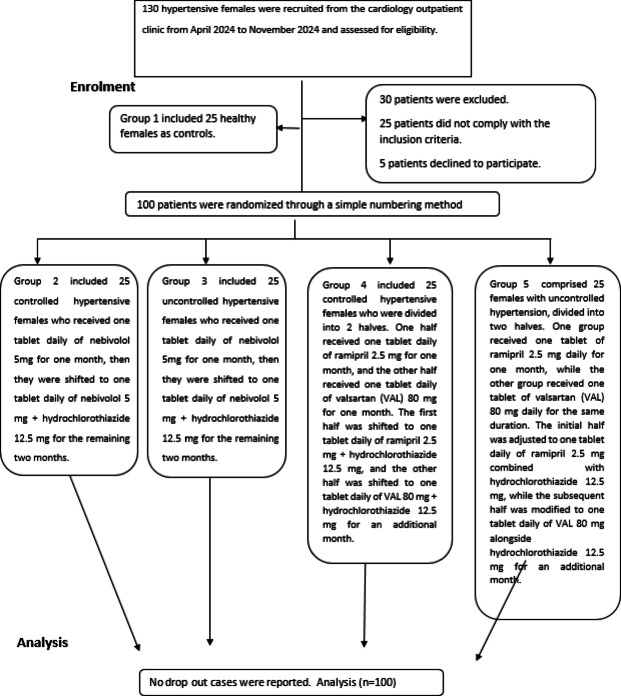
A flowchart demonstrates the methodology of the study.

A prospective randomized controlled study was conducted in the outpatient clinics of the Cardiology Department from April 2024 to November 2024 (Fig. 1). One hundred premenopausal patients and 25 healthy, age-matched females were recruited after providing written informed consent. The study prospectively received approval from the research ethics committee of Beni Suef University on 3rd of March 2024 under the following registration number (IORG0006240/FWA00015574) in accordance with the Helsinki Declaration (2013)^21^. Additionally, the study was prospectively registered in the UMIN registry clinical trials under the following registration number (UMIN000053844) at 13/3/2024 (https://center6.umin.ac.jp/cgi-bin/ctr_e/ctr_view.cgi?recptno=R000061425).

### Inclusion criteria of the patients

Premenopausal participants diagnosed with HTN and reporting a stable partner relationship for the preceding year were recruited. Uncontrolled hypertensive patients who were included in the study were diagnosed according to the European Society of Cardiology guidelines (2024)^22^. The 2024 Guidelines define HTN as a confirmed office systolic blood pressure (BP) of ≥ 140 mmHg or diastolic BP of ≥ 90 mmHg^22^. Confirmation of diagnosis is achieved by out-of-office measurements (Home-based blood pressure measurement (HBPM) or ambulatory blood pressure measurement (ABPM) or at least one repeat office measurement at a subsequent visit. Where screening office BP is 140–159/90–99 mmHg, it is recommended that the diagnosis of HTN should be based on out-of-office BP measurement with ABPM and/or HBPM^22^.

If these measurements were not logistically or economically feasible, then diagnosis could be confirmed on repeated office BP measurements on more than one visit^22^.

### Exclusion criteria of the patients

Pregnant women, breastfeeding women, patients with diabetes mellitus, obesity (body mass index ≥ 30), smoking, history of breast cancer or thromboembolic diseases, major cardiovascular disease, or neurological disorder were excluded. Also, postmenopausal women or female patients with irregular menses, polycystic ovary syndrome or depression, or females with urinary incontinence were excluded from the study. Finally, cases of secondary HTN were excluded by the routine labs that were perfomed to the patients included in the study^22^.

### Inclusion criteria of the controls

Participants included age-matched, healthy females accompanying patients who consented to participate.

### Randomization

All patients were randomized using a simple numbering method. Data were sent in closed envelopes that were opened by an assigned physician to ensure proper allocation concealment. Notably, enrolled cases exhibited no contraindications to the antihypertensive medications utilized, permitting randomization by the simple numbering method into four groups.

### Interventions

A total of 130 participants were screened for eligibility in the study. 25 patients did not comply with the inclusion criteria and 5 patients declined to participate. Thus, a total of 100 participants were randomized into four groups using a simple numbering method.

It should be mentioned that no drop-outs were reported in the current study. Furthermore, all the participants were instructed to do strict contraception due to higher risk of adverse fetal outcomes, including malformations and stillbirths in women exposed to ACEIs or ARBs during early pregnancy, compared to non-exposed controls^23^. Nevertheless, it is worth mentioning that the five major drug classes with evidence for BP-mediated reduction in cardiovascular events are ACEIs, ARBs, dihydropyridine calcium channel blockers (CCBs), diuretics, and BBs). The first four drugs are recommended as first-line options for starting HTN treatment in the general population^22^. Group (1) comprised 25 healthy females designated as controls. Groups (2) and (3) included controlled and uncontrolled hypertensive patients who were administered one tablet of 5 mg nebivolol daily for a duration of one month. The dosage was adjusted to one tablet daily of nebivolol 5 mg combined with hydrochlorothiazide 12.5 mg for the subsequent two months. Groups (4) and (5) consisted of controlled and uncontrolled hypertensive females who received ACEIs/ARBs. Half of the patients in each group were administered one tablet daily of ramipril 2.5 mg for one month, while the other half received one tablet daily of valsartan (VAL) 80 mg for the same duration. After one month, the subjects were transitioned to a daily regimen of one tablet of ramipril 2.5 mg, combined with hydrochlorothiazide 12.5 mg, and one tablet of VAL 80 mg, combined with hydrochlorothiazide 12.5 mg, for two months, respectively. Hydrochlorothiazide was prescribed for 2 months to patients with uncontrolled HTN following one month of the initiation of BBs and ACEIs/ARBs. However, hydrochlorothiazide was also prescribed for controlled hypertensive females receiving BBs and ACEIs/ARBs for two months at the same dose administered for the uncontrolled hypertensive females for homogeneity purpose.

This regimen was presecribed according to the ESC (2024) guidelines which stated that HTN treatment necessitated more than one BP-lowering medication for many patients^22^. Multi-drug therapy from different drug classes could have additive or synergistic effects and led to greater BP reduction than increasing the dose of a monotherapy^22^. The superior BP-lowering efficacy of multi-drug therapy is mediated, at least in part, by the potential of multi-drug therapy to target multiple pathophysiological pathways contributing to perturbed BP in each patient^22^. A further benefit of multi-drug therapy is the potential to use lower doses of each individual BP-lowering agent, which may reduce the side effects and improve long-term compliance^22^. Notably, the diagnosis of HTN was based on self-reports from the patients, who presented with continuous headaches and papilledema, alongside a family history of HTN and no prior treatment for their symptoms. Therefore, during the initial visit, BP was measured using a standard mercury sphygmomanometer in the seated position on two separate days at the same time to confirm the diagnosis of HTN. Furthermore, BP measurements were collected from each patient during follow-up visits. Ideally, BP should be treated to target within 1 month to 3 months, the treatment target in the ESC (2024) Guidelines is always 120–129/70–79 mmHg^22^. After treatment initiation, the patient should be seen frequently until BP is controlled. BP should be controlled, preferably within 1 month to 3 months^22^. The recommended follow-up after 1–3 months not only allows for assessment of tolerance/safety but also allows enough time to gauge the full BP-lowering effect of each drug^22^. The magnitude of BP reduction achieved with the main classes of BP-lowering medications (ACEIs, ARBs, dihydropyridine CCBs, diuretics, and beta-blockers) as monotherapy was similar overall^22^. BP reduction with standard doses of any of these five classes could be expected to be approximately 9/5 mmHg with office BP^22^.

### Outcome measures

At baseline and following 12 weeks of active treatment for HTN, patients completed the validated Arabic version of the Female Sexual Function Index (ArFSFI) during a structured interview^24^. Patients were assessed for anxiety levels using the validated Arabic version of the Generalized Anxiety Disorder Scale (GAD-7) during the structured interview^25,26^. On the second day of menstruation, 5 ml of blood was collected in the early morning for the measurement of serum total testosterone using ELISA kits HUFI03346 (adult women: 15–70 ng/dl), free testosterone with ELISA kits HUFI03346 (adult women: 1.0-8.5 pg/ml), and serum estradiol utilizing ELISA kits HUFI03346 (adult women: 1.0-8.5 pg/ml) and serum estradiol using ELIZA kits UNFI0011 (adult females, non-pregnant, early Follicular phase: 20–150 pg/mL, late follicular phase: 40–350 pg/mL, mid-cycle phase peak: 150–750 pg/mL, luteal phase: 30–450 pg/mL) at baseline and after 12 weeks of active treatment. Routine labs were performed to the patients including fasting blood glucose and glycosylated Hb if fasting blood glucose was elevated. Lipids profile, blood sodium and potassium, haemoglobin and/or haemoticrit, calcium, and TSH were performed. Screening for secondary HTN was conducted by excluding cases of primary aldosteronism, Cushing’s disease, polycythaemia, hyperparathyroidism, and hyperthyroidism.

Blood creatinine and eGFR; urinalysis were performed, and 12-lead ECG was done to assess HMOD (left atrial enlargement, left ventricular hypertrophy) and irregular pulse.

### Statistical Analysis

The data were analyzed using SPSS software version 24. Qualitative data were expressed as numbers and percentages within each group. The normality of quantitative data was assessed using the Kolmogorov-Smirnov test, with results expressed as mean ± standard deviation or median and range. The uniformity of pretreatment values of BP was evaluated by the χ^2^ test. The differences between the two groups were compared using the Student *t*-test for quantitative variables and nonparametric tests (signed rank test and Wilcoxon two-sample test) for the other variables. A *P*-value of < 0.05 was considered significant.

Sample size determination.

The following equation was used n = Z² * P*Q/d2 (n = required sample size, Z = confidence level at 95% (standard value of 1.96), P = estimated prevalence of FSD in hypertensive females based on literature was 8.9% aged 18 to 44^27^, Q = 1-P, d = margin of error (0.05). The calculation yielded n = Z² x PQ/d2= (1.96)2 * (0.089) *(1-0.089) / (0.05)^2^ ≈ 125. Consequently, 100 hypertensive patients and 25 healthy normotensive females were recruited for this study.

## Results

No significant age difference was observed among the participant groups (*p* = 0.82). Mean ages were 39.9 ± 2.5 years for controlled hypertensive patients receiving BBs, 38.8 ± 4.5 years for uncontrolled hypertensive patients receiving BBs, 38.1 ± 2.8 years for controlled hypertensive patients receiving ACEIs/ARBs, and 40.15 ± 3.8 years for uncontrolled hypertensive patients receiving ACEIs/ARBs.

**Fig. 2:**
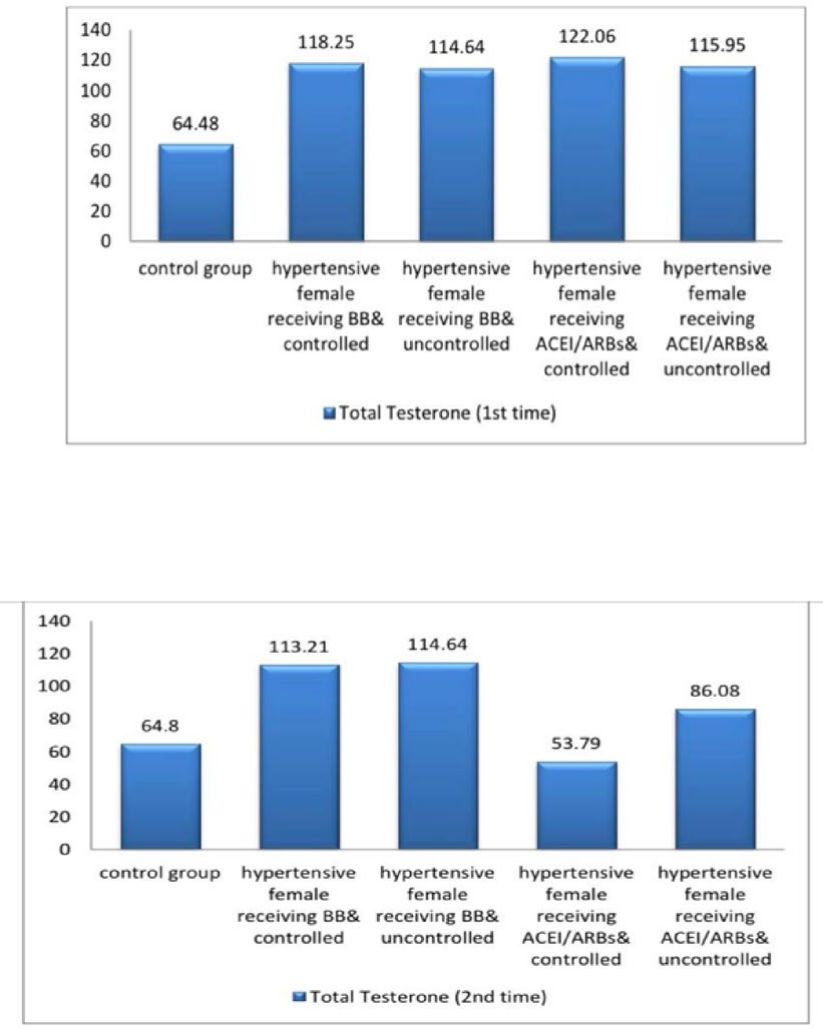
A bar diagram shows total testoseterone among the participants at baseline and after 3 months.

**Fig. 3:**
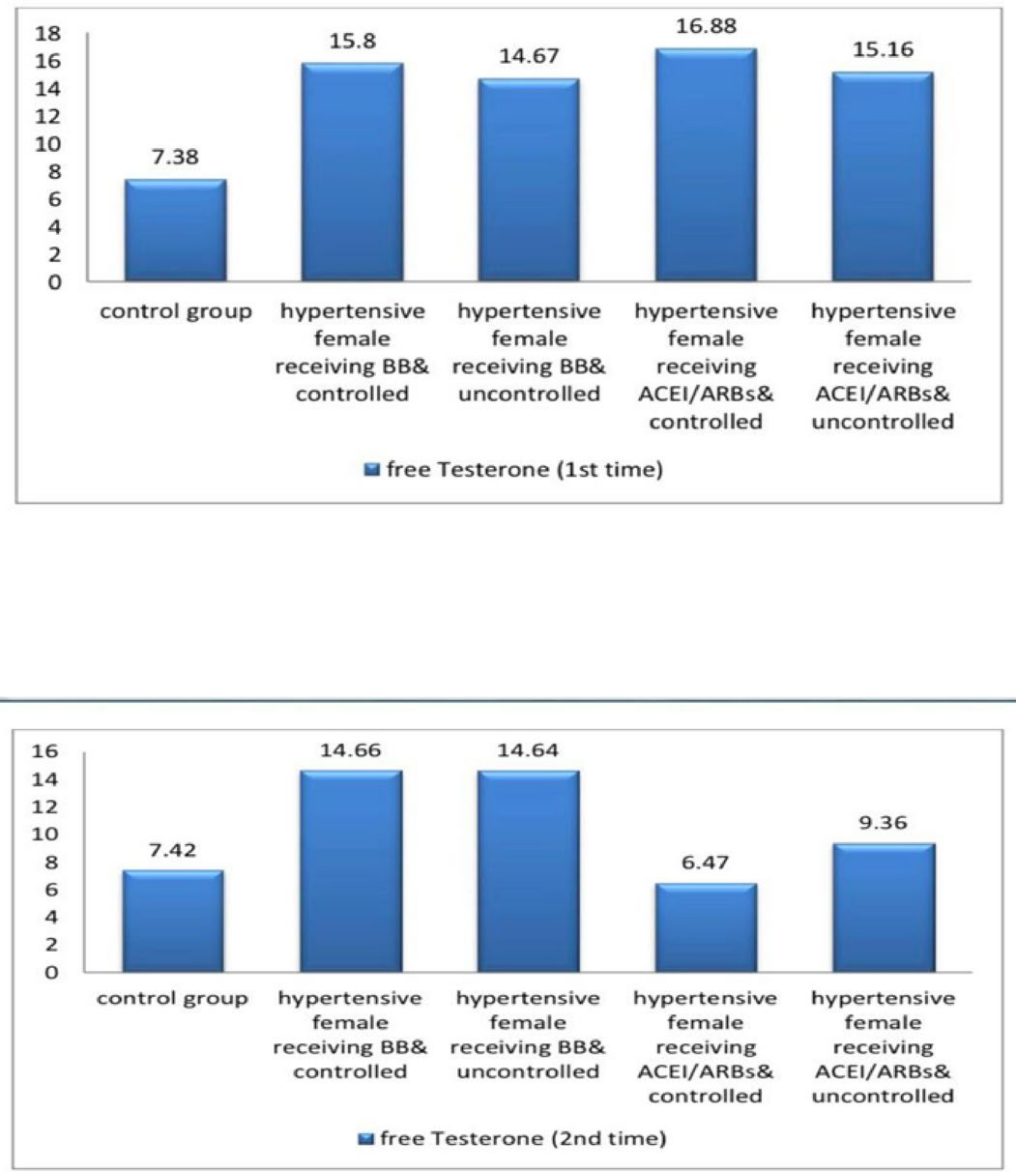
A bar diagram shows free testoseterone among the participants at baseline and after 3 months.

**Fig. 4:**
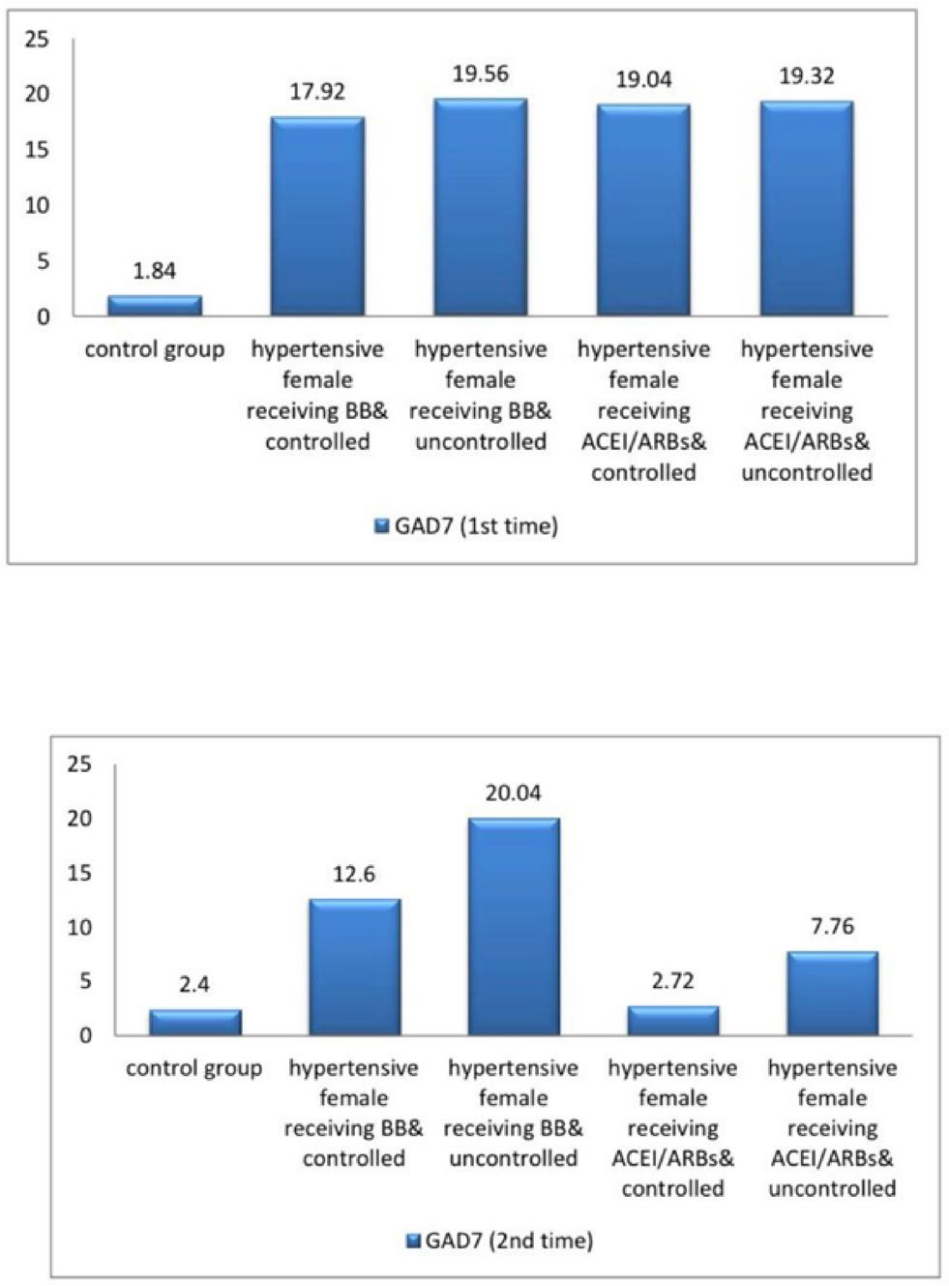
A bar diagram shows GAD score among the participants at baseline and after 3 months.

**Fig. 5:**
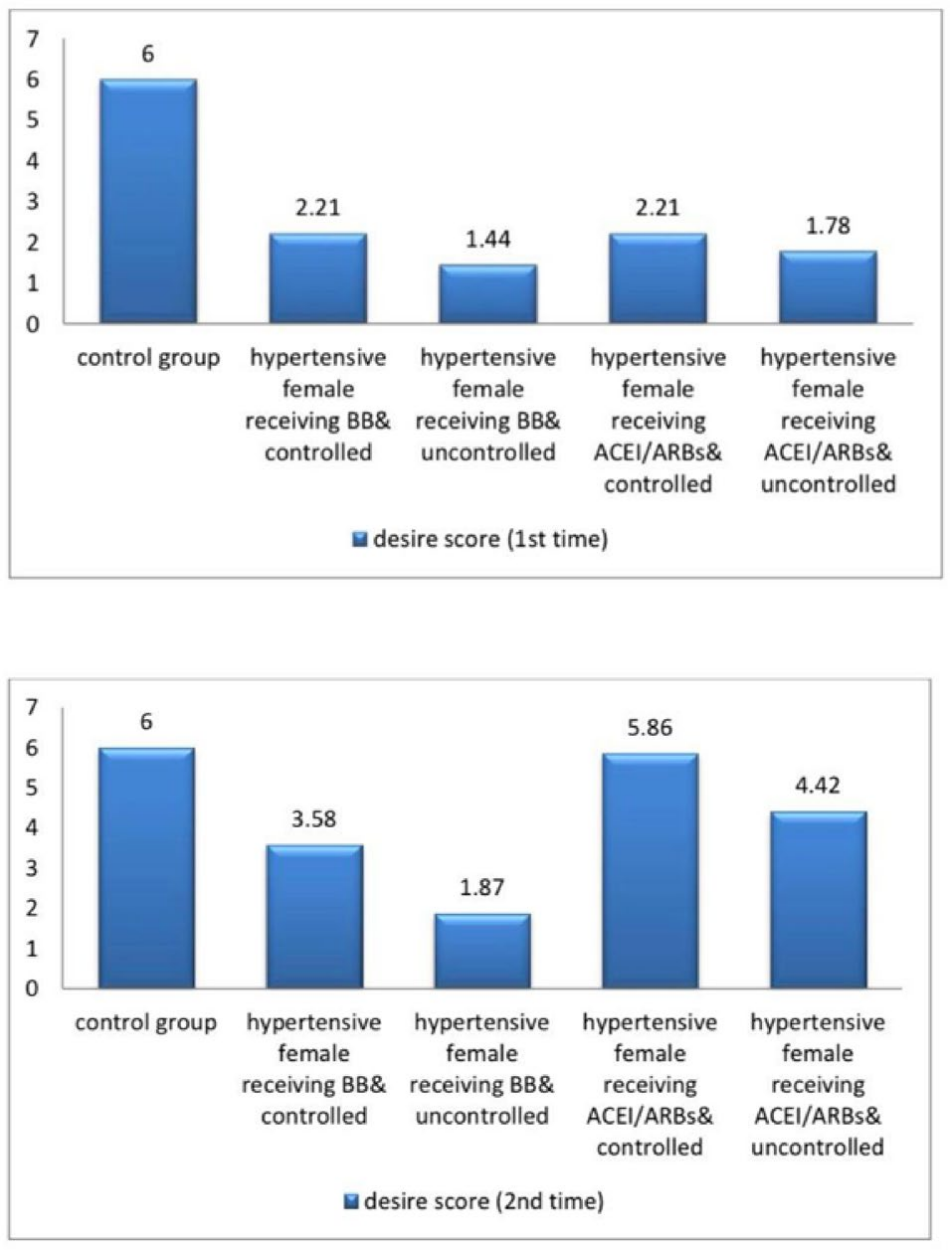
A bar diagram shows desire score among the participants at baseline and after 3 months.

**Fig. 6:**
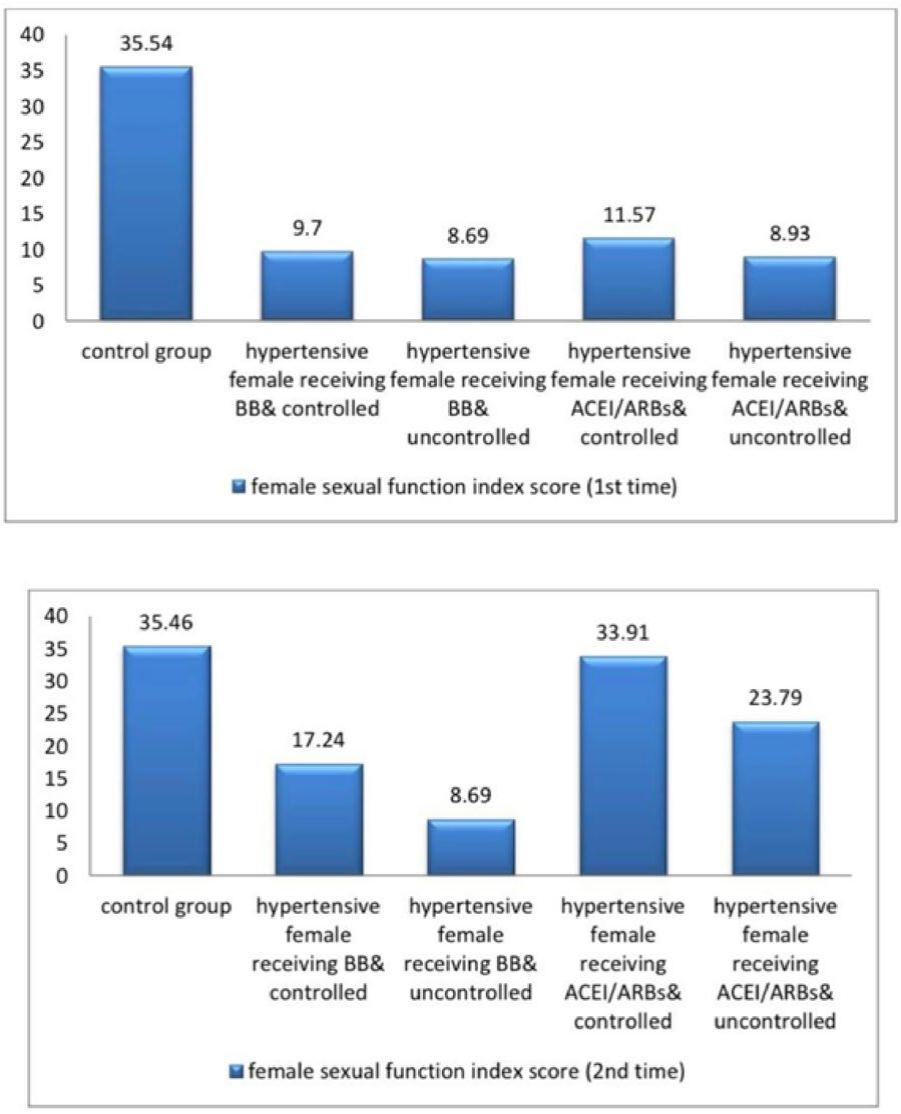
A bar diagram shows the validated Arabic index of female sexual function score among the participants at baseline and after 3 months.

**Fig. 7:**
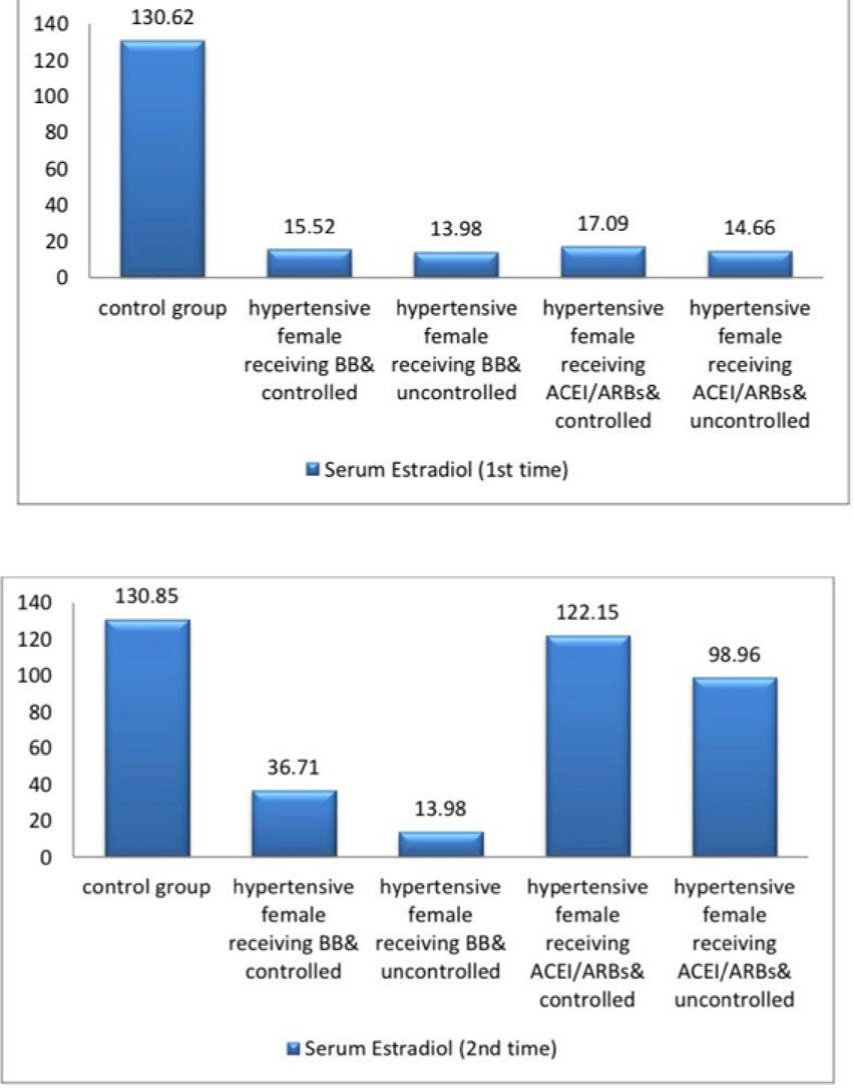
A bar diagram shows estradiol among the participants at baseline and after 3 months.

The current study demonstrated significant differences in baseline systolic blood pressure (SBP) and diastolic blood pressure (DBP) and all female sexual function (FSF) domains and GAD-7 scores, total testosterone, free testosterone levels, and serum estradiol between participants and controls at baseline and after three months (Tables 1 and 2; Figs. 2, 3, 4, 5, 6 and 7). Controlled hypertensive patients receiving BBs exhibited significant decreases in serum total testosterone and free testosterone alongside a significant increase in GAD-7 score (*p* < 0.05). Conversely, they demonstrated significant increases in serum estradiol and total female sexual function score, with improvements across all female sexual function domains except pain (*p* < 0.05; Table 3). Uncontrolled hypertensive patients receiving BBs showed significant increases in desire, arousal, orgasm, and satisfaction scores (*p* < 0.05), though these remained lower than corresponding scores in controlled hypertensive patients. Their total female sexual function score remained unchanged (*p* > 0.05), while GAD-7 scores increased significantly (Table 3). Moreover, controlled hypertensive females receiving ACEI/ARBs demonstrated significant decreases in serum total testosterone and free testosterone and GAD-7 score after 3 months and showed significant increases in all FSF domains and total FSD score and serum estradiol after 3 months (Table 4).

**Table 1:** shows baseline systolic blood pressure and diastolic blood pressure and total and free testosterone and estradiol and female sexual function domains and GAD7 among the participants.

*N* = 100	Controls (*n* = 25)	Controlled hypertensive females receiving BBs (*n* = 25)	Uncontrolled hypertensive females receiving BBs (*n* = 25)	Controlled hypertensive females receiving ACEI/ARBs (*n* = 25)	Uncontrolled hypertensive female receiving ACEI/ARBs (*n* = 25)	*P* value
Systolic blood Pressure	115.6 ± 6.51	142.4 ± 5.23 ^*^	166.40 ± 14.11 ^*^	138.8 ± 7.81 ^*$^	172.8 ± 14.29 ^*^	< 0.001
Diastolic blood pressure	77.2 ± 4.58	78.4 ± 6.24	103.20 ± 4.54 ^*^	79.6 ± 4.55 ^$^	103.8 ± 4.63 ^*^	< 0.001
Total testosterone	64.48 ± 3.72	118.25 ± 2.46 ^*^	114.64 ± 3.07 ^*#^	122.06 ± 3.13 ^*#$^	115.95 ± 3.27^*@^	< 0.001
Free testosterone	7.38 ± 0.44	15.8 ± 0.85 ^*^	14.67 ± 0.97 ^*#^	16.88 ± 0.81 ^*#$^	15.16 ± 1.18 ^*@^	< 0.001
Serum estradiol	130.62 ± 5.01	15.52 ± 1.11 ^*^	13.98 ± 1.28 ^*^	17.09 ± 1.2 ^*$^	14.66 ± 1.51 ^*@^	< 0.001
Desire score	6 ± 0	2.21 ± 0.45 ^*^	1.44 ± 0.49 ^*#^	2.21 ± 0.45 ^*$^	1.78 ± 0.61 ^*#@^	< 0.001
Arousal score	5.93 ± 0.16	1.63 ± 0.59 ^*^	1.25 ± 0.24 ^*#^	1.97 ± 0.59 ^*$^	1.39 ± 0.45 ^*@^	< 0.001
Lubrication score	5.93 ± 0.13	1.3 ± 0.33 ^*^	1.2 ± 0 ^*^	1.63 ± 0.59 ^*#$^	1.24 ± 0.18 ^*@^	< 0.001
Orgasm score	5.96 ± 0.23	1.58 ± 0.57 ^*^	1.58 ± 0.57 ^*^	1.92 ± 0.6 ^*^	1.25 ± 0.24 ^*@^	< 0.001
Satisfaction score	5.94 ± 0.15	1.78 ± 0.61 ^*^	2.02 ± 0.57 ^*^	2.35 ± 0.24 ^*#^	2.08 ± 0.48 ^*^	< 0.001
Pain score	5.81 ± 0.23	1.2 ± 0 ^*^	1.2 ± 0 ^*^	1.49 ± 0.52 ^*#$^	1.2 ± 0 ^*@^	< 0.001
Female sexual function index score	35.54 ± 0.57	9.7 ± 1.03 ^*^	8.69 ± 1.11 ^*#^	11.57 ± 1.42 ^*#$^	8.93 ± 1.23 ^*@^	< 0.001
GAD 7 score	1.84 ± 0.85	17.92 ± 2.1 ^*^	19.56 ± 1.04 ^*#^	19.04 ± 1.4 ^*^	19.32 ± 1.28 ^*#^	< 0.001

N.B. *: statistically significant compared to corresponding value in controls (*P* < 0.05).

#: statistically significant compared to corresponding value in hypertensive female receiving BB & controlled group (*P* < 0.05).

$: statistically significant compared to corresponding value in hypertensive female receiving BB & uncontrolled group (*P* < 0.05).

@: statistically significant compared to corresponding value in hypertensive female receiving ACEI/ARBs & controlled group (*P* < 0.05).

BBs= betablockers; ACEIs/ARBs = angiotensin converting enzyme inhibitors/ angiotensin receptor blockers; GAD 7 = validated Arabic version of generalized anxiety disorder scale.

**Table 2:** shows systolic blood pressure and diastolic blood pressure and total and free testosterone and estradiol and female sexual function domains and GAD7 among the participants after 3 months.

*N* = 100	Controls (*N* = 25)	Controlled hypertensive females receiving BBs (*N* = 25)	Uncontrolled hypertensive females receiving BBs (*N* = 25)	Controlled hypertensive females receiving ACEI/ARBs (*N* = 25)	Uncontrolled hypertensive female receiving ACEI/ARBs (*N* = 25)	*P* value
Systolic blood pressure	112.8 ± 7.37	116.4 ± 4.9	172.80 ± 14.29^*#^	116.8 ± 4.76 ^$^	173.2 ± 14.35 ^*#@^	< 0.001
Diastolic blood pressure	76 ± 5	75.2 ± 5.1	103.80 ± 4.63^*#^	76 ± 5 ^$^	103.2 ± 4.54 ^*#@^	0.002
Total testosterone	64.8 ± 3.56	113.21 ± 2.53 ^*^	114.64 ± 3.08 ^*^	53.79 ± 4.78 ^*#$^	86.08 ± 3.2 ^*#$@^	< 0.001
Free testosterone	7.42 ± 0.36	14.66 ± 0.71 ^*^	14.64 ± 0.98 ^*^	6.47 ± 0.5 ^*#$^	9.36 ± 0.58 ^*#$@^	< 0.001
Serum estradiol	130.85 ± 4.9	36.71 ± 4.83 ^*^	13.98 ± 1.32 ^*#^	122.15 ± 2.98 ^*#$^	98.96 ± 18.8 ^*#$@^	< 0.001
Desire score	6 ± 0	3.58 ± 0.12 ^*^	1.87 ± 0.61 ^*#^	5.86 ± 0.4 ^#$^	4.42 ± 0.57 ^*#$@^	< 0.001
Arousal score	5.93 ± 0.16	3.23 ± 0.56 ^*^	1.44 ± 0.49 ^*#^	5.75 ± 0.32 ^#$^	4.24 ± 0.6 ^*#$@^	< 0.001
Lubrication score	5.94 ± 0.13	2.86 ± 0.55 ^*^	1.2 ± 0 ^*#^	5.75 ± 0.32 ^#$^	3.86 ± 0.44 ^*#$@^	< 0.001
Orgasm score	5.9 ± 0.21	2.62 ± 0.38 ^*^	1.63 ± 0.59 ^*#^	5.47 ± 0.46 ^*#$^	3.87 ± 0.38 ^*#$@^	< 0.001
Satisfaction score	5.95 ± 0.13	2.4 ± 0 ^*^	1.34 ± 0.4 ^*#^	6 ± 0 ^#$^	3.79 ± 0.29 ^*#$@^	< 0.001
Pain score	5.76 ± 0.26	2.4 ± 0 ^*^	1.2 ± 0 ^*#^	5.15 ± 0.31 ^*#$^	3.62 ± 0.08 ^*#$@^	< 0.001
Female sexual function index score	35.46 ± 0.54	17.24 ± 0.98 ^*^	8.69 ± 1.11 ^*#^	33.91 ± 1.47 ^*#$^	23.79 ± 1.56 ^*#$@^	< 0.001
GAD7	2.4 ± 1	12.6 ± 1.12 ^*^	20.04 ± 0.89 ^*#^	2.72 ± 0.74 ^#$^	7.76 ± 0.72 ^*#$@^	< 0.001

N.B. *: statistically significant compared to corresponding value in controls (*P* < 0.05).

#: statistically significant compared to corresponding value in hypertensive female receiving BB & controlled group (*P* < 0.05).

$: statistically significant compared to corresponding value in hypertensive female receiving BB & uncontrolled group (*P* < 0.05).

@: statistically significant compared to corresponding value in hypertensive female receiving ACEI/ARBs & controlled group (*P* < 0.05).

BBs= betablockers; ACEIs/ARBs = angiotensin converting enzyme inhibitors/ angiotensin receptor blockers; GAD 7 = validated Arabic version of generalized anxiety disorder scale.

**Table 3:** compares between controlled hypertensive females receiving BBs and uncontrolled hypertensive females receiving BB at baseline and after 3 months.

N = 50	Controlled hypertensive females receiving BB (N = 25)				P value	Uncontrolled hypertensive females receiving BB (N = 25)				P value
	Baseline		After 3 months			Baseline		After 3 months		
	Mean	SD	Mean	SD		Mean	SD	Mean	SD	
Systolic blood pressure	142.40	5.23	116.40	4.90	< 0.001	152.00	7.07	145.60	5.07	< 0.001
Diastolic blood pressure	78.40	6.24	75.20	5.10	0.018	84.40	5.07	80.80	5.72	0.026
Total testosterone	118.25	2.46	113.21	2.53	< 0.001	114.64	3.07	114.64	3.08	0.765
Free testosterone	15.80	0.85	14.66	0.71	< 0.001	14.67	0.97	14.64	0.98	0.302
Serum estradiol	15.52	1.11	36.71	4.83	< 0.001	13.98	1.28	13.98	1.32	0.914
Desire score	2.21	0.45	3.58	0.12	< 0.001	1.44	0.49	1.87	0.61	0.001
Arousal score	1.63	0.59	3.23	0.56	< 0.001	1.25	0.24	1.44	0.49	0.043
Lubrication score	1.30	0.33	2.86	0.55	< 0.001	1.20	0.00	1.20	0.00	0.327
Orgasm score	1.58	0.57	2.62	0.38	< 0.001	1.58	0.57	1.63	0.59	< 0.001
Satisfaction score	1.78	0.61	2.40	0.00	< 0.001	2.02	0.57	1.34	0.40	< 0.001
Pain score	1.20	0.00	2.40	0.00	–––	1.20	0.00	1.20	0.00	–––
Female sexual function index score	9.70	1.03	17.24	0.98	< 0.001	8.69	1.11	8.69	1.11	–––
GAD7	17.92	2.10	12.60	1.12	< 0.001	19.56	1.04	20.04	0.89	0.043

N.B. BBs= betablockers; GAD 7 = validated Arabic version of generalized anxiety disorder scale.

**Table 4:** compares between controlled hypertensive females receiving ACEI/ARBs and uncontrolled hypertensive females receiving ACEI/ARBs at baseline and after 3 months.

N = 50	Controlled hypertensive females receiving ACEI/ARBs (N = 25)				P value	Uncontrolled hypertensive females receiving ACEI/ARBs (N = 25)				P value
	Baseline		After 3 months			Baseline		After 3 months		
	Mean	SD	Mean	SD		Mean	SD	Mean	SD	
Systolic blood pressure	138.80	7.81	116.80	4.76	< 0.001	140.00	5.77	137.60	5.23	0.110
Diastolic blood pressure	79.60	4.55	76.00	5.00	0.017	82.00	7.07	77.60	5.23	0.009
Total testosterone	122.06	3.13	53.79	4.78	< 0.001	115.95	3.27	86.08	3.20	< 0.001
Free testosterone	16.88	0.81	6.47	0.50	< 0.001	15.16	1.18	9.36	0.58	< 0.001
Serum estradiol	17.09	1.20	122.15	2.98	< 0.001	14.66	1.51	98.96	18.80	< 0.001
Desire score	2.21	0.45	5.86	0.40	< 0.001	1.78	0.61	4.42	0.57	< 0.001
Arousal score	1.97	0.59	5.75	0.32	< 0.001	1.39	0.45	4.24	0.60	< 0.001
Lubrication score	1.63	0.59	5.75	0.32	< 0.001	1.24	0.18	3.86	0.44	< 0.001
Orgasm score	1.92	0.60	5.47	0.46	< 0.001	1.25	0.24	3.87	0.38	< 0.001
Satisfaction score	2.35	0.24	6.00	0.00	< 0.001	2.08	0.48	3.79	0.29	< 0.001
Pain score	1.49	0.52	5.15	0.31	< 0.001	1.20	0.00	3.62	0.08	< 0.001
Female sexual function index score	11.57	1.42	33.91	1.47	< 0.001	8.93	1.23	23.79	1.56	< 0.001
GAD7	19.04	1.40	2.72	0.74	< 0.001	19.32	1.28	7.76	0.72	< 0.001

N.B. ACEIs/ARBs = angiotensin converting enzyme inhibitors/ angiotensin receptor blockers; GAD 7 = validated Arabic version of generalized anxiety disorder scale.

Meanwhile, uncontrolled hypertensive females receiving ACEI/ARBs demonstrated significant increases in all FSF domains and total FSD scores after three months (Table 4). However, these scores were lower compared to those of their counterparts with controlled HTN (Table 4). Remarkably, their GAD-7 significantly decreased after 3 months (Table 4). The study revealed significant negative correlations between domains of FSF and age, as well as between SBP, DBP, total testosterone, free testosterone, and GAD-7 scores after three months.

Concurrently, there were significant increases in all FSF domains, total FSD scores, and serum estradiol levels after the same period (Table 5). Further analysis revealed significant positive correlations between SBP and DBP and total testosterone and free testosterone and GAD-7 score at baseline (Table 6). In contrast, a significant negative correlation between SBP and DBP and serum estradiol was observed at baseline (Table 6). After three months, significant positive correlations between SBP and total testosterone and free testosterone and GAD-7 score were observed (Table 6). Meanwhile, a significant negative correlation between SBP and serum estradiol was noted after 3 months (Table 6). DBP demonstrated a significant positive correlation with GAD-7 and a significant negative correlation with serum estradiol after 3 months (Table 6). Notably, no correlation was observed between DBP and either total or free testosterone levels at the 3-month assessment (Table 6).

**Table 5:** shows correlations between domains of female sexual function and the different variables in the study at baseline and after 3 months among female patients.

*N* = 100		Desire score	Arousal score	Lubrication score	Orgasm score	Satisfaction score	Pain score	Female sexual function index score
Age	r	-0.856-	-0.847-	-0.820-	-0.820-	-0.826-	-0.795-	-0.847-
	P value	< 0.001	< 0.001	< 0.001	< 0.001	< 0.001	< 0.001	< 0.001
Systolic blood pressure (baseline)	r	-0.858-	-0.836-	-0.839-	-0.796-	-0.825-	-0.802-	-0.845-
	P value	< 0.001	< 0.001	< 0.001	< 0.001	< 0.001	< 0.001	< 0.001
Diastolic blood pressure (baseline)	r	-0.314-	-0.293-	-0.259-	-0.287-	-0.196-	-0.256-	-0.274-
	P value	< 0.001	< 0.001	0.004	0.001	0.028	0.004	0.002
Total testosterone (baseline)	r	-0.899-	-0.916-	-0.949-	-0.919-	-0.914-	-0.963-	-0.949-
	P value	< 0.001	< 0.001	< 0.001	< 0.001	< 0.001	< 0.001	< 0.001
Free testosterone (baseline)	r	-0.830-	-0.853-	-0.902-	-0.867-	-0.852-	-0.922-	-0.892-
	P value	< 0.001	< 0.001	< 0.001	< 0.001	< 0.001	< 0.001	< 0.001
Serum estradiol (baseline)	r	0.957	0.967	0.985	0.964	0.960	0.989	0.993
	P value	< 0.001	< 0.001	< 0.001	< 0.001	< 0.001	< 0.001	< 0.001
GAD 7 score (baseline)	r	-0.956-	-0.966-	-0.965-	-0.935-	-0.928-	-0.963-	-0.975-
	P value	< 0.001	< 0.001	< 0.001	< 0.001	< 0.001	< 0.001	< 0.001
Age	r	-0.737-	-0.745-	-0.748-	-0.758-	-0.703-	-0.746-	-0.752-
	P value	< 0.001	< 0.001	< 0.001	< 0.001	< 0.001	< 0.001	< 0.001
Systolic blood pressure (after 3 months)	r	-0.676-	-0.664-	-0.672-	-0.617-	-0.623-	-0.641-	-0.663-
	P value	< 0.001	< 0.001	< 0.001	< 0.001	< 0.001	< 0.001	< 0.001
Diastolic blood pressure (after 3 months)	r	-0.261-	-0.276-	-0.261-	-0.198-	-0.218-	-0.222-	-0.243-
	P value	0.003	0.002	0.003	0.027	0.015	0.013	0.006
Total testosterone (after 3 months)	r	-0.868-	-0.869-	-0.906-	-0.903-	-0.958-	-0.925-	-0.922-
	P value	< 0.001	< 0.001	< 0.001	< 0.001	< 0.001	< 0.001	< 0.001
Free testosterone (after 3months)	r	-0.846-	-0.850-	-0.882-	-0.887-	-0.935-	-0.916-	-0.902-
	P value	< 0.001	< 0.001	< 0.001	< 0.001	< 0.001	< 0.001	< 0.001
Serum estradiol (after 3 months)	r	0.925	0.933	0.944	0.941	0.952	0.956	0.958
	P value	< 0.001	< 0.001	< 0.001	< 0.001	< 0.001	< 0.001	< 0.001
GAD 7 score (after 3 months)	r	-0.958-	-0.959-	-0.965-	-0.938-	-0.960-	-0.966-	-0.976-
	P value	< 0.001	< 0.001	< 0.001	< 0.001	< 0.001	< 0.001	< 0.001

N.B. GAD 7 = validated Arabic version of generalized anxiety disorder scale.

**Table 6:** shows correlations between SBP and DBP and total testosterone and free testosterone and estradiol and GAD 7 score at baseline and after 3 months among female patients.

*N* = 100		Total testosterone	Free testosterone	Serum estradiol	GAD 7
Systolic blood pressure (baseline)	r	0.748	0.668	-0.822-	0.806
	P value	< 0.001	< 0.001	< 0.001	< 0.001
Diastolic blood pressure (baseline)	r	0.226	0.202	-0.263-	0.298
	P value	0.011	0.024	0.003	0.001
Systolic blood pressure (after 3 months)	r	0.496	0.410	-0.505-	0.662
	P value	< 0.001	< 0.001	< 0.001	< 0.001
Diastolic blood pressure (after 3 months)	r	0.164	0.133	-0.209-	0.257
	P value	0.067	0.138	0.019	0.004

N.B. GAD 7 = validated Arabic version of generalized anxiety disorder scale; SBP= Systolic Blood Pressure; DBP= Diastolic Blood Pressure.

## Discussion

The mechanism of male sexual dysfunction due to hypertension and antihypertensive medications has been extensively documented in numerous studies, whereas the mechanism of FSD remains less understood. Despite its prevalence, it remains largely unexplained, potentially due to the absence of effective treatment^28^. Notably, there is a limited number of studies on the mechanism and effects of BBs as antihypertensives in FSF. However, females with FSD should receive the same antihypertensive treatment as male patients, as the literature indicates comparable effects on sexual function in both sexes^29^. Doumas et al. (2006) demonstrated an increased prevalence of FSD among hypertensive females due to the intake of nonselective BBs^30^. Another explanation for the negative impact of nonselective BBs on FSF is the testosterone-lowering effect in women^31^.

The remodeling of blood vessel walls due to elevated blood pressure leads to impaired vascular supply to the clitoris and vagina, resulting in vasculogenic FSD^30,31^. NO is essential for the relaxation of smooth muscles in the tunica media, leading to increased blood flow to the genitalia, which parallels the process of erection in males during sexual arousal in females^30,31^. HTN could lead to FSD because of impaired NO bioavailability^27^. Consistently, antihypertensive that acts on the NO pathway ameliorates FSF^19^. Nebivolol is a third-generation BB that exhibits vasodilating effects through its ability to release NO from endothelial cells, in addition to its beta-adrenergic receptor-blocking activity^19^. Two prior trials confirmed the hypothesis that ARBs improved FSF^32,33^. Furthermore, Fogari et al. (2004) reported that valsartan administration over a period of 4 months enhanced sexual desire and fantasy in 50 females with HTN^32^. In a similar trend, hypertensive females receiving losartan for three months exhibited increased arousal^34^.

Similarly, the use of felodipine–irbesartan for one year resulted in improved sexual function^33^. The positive effect of ARB drugs on FSF can be ascribed to conversely, four prior studies found no association between ACEI/ARB and FSD^30–31,35−36^. The current study found no significant adverse effects of thiazides on FSF. Several studies have similarly found no association between diuretics and FSD^30–31,35−38^. Conversely, Okeahialam and colleagues identified the highest ratio of FSD in females receiving thiazide antihypertensive^39^. However, this observation was not significant, potentially due to the small sample size^39^. The study indicated that the primary complaint associated with FSD was reduced libido^39^. Thiazides may influence sexual function through mechanisms such as decreased blood volume and hyperlipidemia^1^. However, uncertainty persists regarding the potential detrimental effects of diuretics on FSF^1^.

Healthy females in this study exhibited the highest levels of E2, alongside the lowest total and free testosterone levels, in comparison to the patients. Additionally, both controlled and uncontrolled hypertensive patients treated with ACEI/ARBs exhibited notable reductions in total testosterone and free testosterone levels, alongside a significant rise in E2, when compared to their counterparts receiving ACEI/ARBs. As previously explained, E2 plays a crucial role in sexual behaviour in the females because of high concentrations in the brain^5,9^. Moreover, Estrogen regulates vascular function through genomic and non-genomic mechanisms^40^ as well as improving vaginal lubrication through improving peripheral blood flow and peripheral nerve function^41^. Noteworthy, estrogen status infleunces the sexual response because of it’s link with the sensitivity of genital and non-genital skin^5^. The present study identified significant positive correlations between baseline SBP and SBP after three months with total and free testosterone levels and GAD-7 scores, alongside significant negative correlations with E2 levels. At baseline, DPB demonstrated significant positive correlations with total and free testosterone levels as well as GAD-7 scores, while exhibiting significant negative correlations with estradiol. After three months, DPB showed significant positive correlations with estradiol and GAD-7 scores; however, no correlation was observed with testosterone, both total and free. Gonadal steroids, especially estrogen, play a vital role in maintaining vascular health and insulin sensitivity in both sexes^42^. E2 is considered a key factor in maintaining a positive cardiovascular profile in premenopausal women^43^. The mechanism through which androgens elevate blood pressure is not fully elucidated^44^. Moreover, polycystic ovary syndrome is associated with obesity and a higher risk of cardiovascular disease attributed to high levels of androgens in women^45,46^.

Furthermore, while testosterone has been proposed as a significant moderator of women’s sexual desire and behavior, the effects of androgens in women relative to men remain inadequately understood^47–50^. Additionally, free testosterone is more significant than total testosterone because it can cross the blood-brain barrier to activate specific brain structures associated with sexual function, including the hypothalamus, pituitary, and amygdala^47–50^. This finding aligns with the current study’s results, indicating that free testosterone levels in healthy females are comparable to those in uncontrolled hypertensive patients treated with ACEI/ARBs and higher than in controlled hypertensive patients receiving ACEIs/ARBs. Additionally, estradiol fluctuations may modulate female sexual desire^51^. A critical systematic review and meta-analysis established that endogenous E2 exhibited a stronger association with libido in eumenorrheic and perimenopausal females than supraphysiological testosterone supplementation in prior therapeutic studies^52^. Notably, GAD-7 scores showed significant negative correlations with all domains of the ArFSFI. In contrast, Nascimento et al. (2015) did not establish a relationship between anxiety and depression across various domains of FSF despite participants exhibiting elevated levels of anxiety and depression^13^. This study represents one of the limited investigations comparing antihypertensive impacts on female sexual function in premenopausal patients, demonstrating the superior efficacy of ACEIs/ARBs over alternative agents. Menopause and other medical comorbidities, such as dyslipidemia and diabetes mellitus, were excluded from the current study due to their potential impact on sexual functions in both females and males^15,53^. Postmenopausal women experience vaginal dryness due to decreased estrogen levels, which is exacerbated by inadequate pelvic blood perfusion, leading to a decline in FSF^15^.

Accordingly, Cipriani & Simon recommended screening of postmenopausal women for cardiovascular risk factors and FSD^54^. Several methodological limitations warrant acknowledgment. Firstly, the modest sample size constrains generalizability. Secondly, the relatively brief follow-up period may limit the detection of long-term effects. Additionally, the systematic addition of thiazides across all intervention groups introduces potential heterogeneity that could bias outcomes. Furthermore, it should be mentioned that multiple domains and correlations were tested without correction for multiplicity and without multivariable adjustment (e.g., for age, BP, BMI within the < 30 range, baseline ArFSFI, or baseline GAD-7), which might raise the risk of type I error. Additionally, the absence of side effects of thiazides on the sexual function of the participants could not be fully elucidated in the current study as there was no true non-thiazide comparator at 3 months. Finally, the absence of progesterone measurements represents a notable limitation, especially considering the baseline variability in estradiol and testosterone levels among participants. This study revealed notable intergroup variations in hormonal profiles after the 3-month intervention. This study explores a significantly overlooked area; therefore, healthcare providers treating women with FSD and/or hypertension should consider the potential pathophysiological connections between these conditions.

## Conclusion

ACIs/ARBs may be preferentially prescribed over BBs for hypertensive patients to mitigate FSD risk. Future large-scale cohort studies are warranted to validate these findings as this study was a single center and of small sample size.

## Supplementary Information

Below is the link to the electronic supplementary material.

Supplementary Material 1

Supplementary Material 2

## Data Availability

The data that supports the findings of this study are available from the corresponding author upon reasonable request.
