# Supplementary material for: Sexual function among controlled and uncontrolled hypertensive females receiving beta-blockers or ACEI/ARB and thiazides: a prospective randomized controlled study
Source: Sci Rep. 2026 Mar 17;16:9227. doi: 10.1038/s41598-026-40790-2 (PMC13000276; doi:10.1038/s41598-026-40790-2)
Supplement: Supplementary file 1 — Supplementary Material 1 [file 41598_2026_40790_MOESM1_ESM.doc]

STROBE Statement—checklist of items that should be included in reports of observational studies

|  | Item No | Recommendation |
| --- | --- | --- |
| **Title and abstract** | 1 | *(a)* Indicate the study’s design with a commonly used term in the title or the abstract  Randomized controlled study as there were controls but they did not receive placebo |
|  |  | (*b*) Provide in the abstract an informative and balanced summary of what was done and what was found  See pages 1 & 2 lines 1-38 |
| Introduction | | |
| Background/rationale | 2 | Explain the scientific background and rationale for the investigation being reported  The impact of antihypertensives on female sexual function was scarcely investigated and a little is known about the interplay between HTN and depression and anxiety as well as testosterone (total and free) and estradiol |
| Objectives | 3 | State specific objectives, including any prespecified hypotheses  See page 4 lines 72-77 |
| Methods | | |
| Study design | 4 | Present key elements of study design early in the paper  See page 2 lines 39-40 |
| Setting | 5 | Describe the setting, locations, and relevant dates, including periods of recruitment, exposure, follow-up, and data collection  See pages 4-5 lines 80-87 |
| Participants | 6 | (*a*) *Case-control study*—Give the eligibility criteria, and the sources and methods of case ascertainment and control selection. Give the rationale for the choice of cases and controls  See page 5-6 lines 88-133 |
|  |  | (*b*) *Case-control study*—For matched studies, give matching criteria and the number of controls per case  *See page 6 lines 142-148* |
| Variables | 7 | Clearly define all outcomes, exposures, predictors, potential confounders, and effect modifiers. Give diagnostic criteria, if applicable  See results section |
| Data sources/ measurement | 8* | For each variable of interest, give sources of data and details of methods of assessment (measurement). Describe comparability of assessment methods if there is more than one group |
| Bias | 9 | Describe any efforts to address potential sources of bias  We added thiazides to all groups, and we excluded postmenopausal females as well as females with PCO or patients suffering from chronic illnesses. Finally obese females were excluded |
| Study size | 10 | Explain how the study size was arrived at  *See page 6 lines 142-148* |
| Quantitative variables | 11 | Explain how quantitative variables were handled in the analyses. If applicable, describe which groupings were chosen and why  See statistical analysis |
| Statistical methods | 12 | (*a*) Describe all statistical methods, including those used to control for confounding  See statistical analysis |
|  |  | (*b*) Describe any methods used to examine subgroups and interactions  See statistical analysis |
|  |  | (*c*) Explain how missing data were addressed  See statistical analysis |
|  |  | (*d*) *Case-control study*—If applicable, explain how matching of cases and controls was addressed  See sample size determination |
|  |  | (*e*) Describe any sensitivity analyses  Quantitative data were tested for normality using Kolmogorov Smirnov test |

Continued on next page

| Results | | |
| --- | --- | --- |
| Participants | 13* | (a) Report numbers of individuals at each stage of study—eg numbers potentially eligible, examined for eligibility, confirmed eligible, included in the study, completing follow-up, and analysed  See flow chart |
|  |  | (b) Give reasons for non-participation at each stage  See flow chart |
|  |  | (c) Consider use of a flow diagram  See flow chart |
| Descriptive data | 14* | (a) Give characteristics of study participants (eg demographic, clinical, social) and information on exposures and potential confounders  we excluded postmenopausal females as well as females with PCO or patients suffering from chronic illnesses. Finally obese females were excluded. Thus, we presented their age only. |
|  |  | (b) Indicate number of participants with missing data for each variable of interest  See statistical analysis |
|  |  | (c) *Cohort study*—Summarise follow-up time (eg, average and total amount)  Not applicable (case-control) |
| Outcome data | 15* |  |
|  |  | *Case-control study—*Report numbers in each exposure category, or summary measures of exposure see sample size determination |
|  |  |  |
| Main results | 16 | (*a*) Give unadjusted estimates and, if applicable, confounder-adjusted estimates and their precision (eg, 95% confidence interval). Make clear which confounders were adjusted for and why they were included see statistical analysis and results |
|  |  | (*b*) Report category boundaries when continuous variables were categorized  see statistical analysis and results |
|  |  | (*c*) If relevant, consider translating estimates of relative risk into absolute risk for a meaningful time period see statistical analysis and results |
| Other analyses | 17 | Report other analyses done—eg analyses of subgroups and interactions, and sensitivity analyses see statistical analysis and results |
| Discussion | | |
| Key results | 18 | Summarise key results with reference to study objectives  See conclusion |
| Limitations | 19 | Discuss limitations of the study, taking into account sources of potential bias or imprecision. Discuss both direction and magnitude of any potential bias  See limitations |
| Interpretation | 20 | Give a cautious overall interpretation of results considering objectives, limitations, multiplicity of analyses, results from similar studies, and other relevant evidence  See discussion |
| Generalisability | 21 | Discuss the generalisability (external validity) of the study results see discussion |
| Other information | | |
| Funding | 22 | Give the source of funding and the role of the funders for the present study and, if applicable, for the original study on which the present article is based  The current study did not receive any fund |

*Give information separately for cases and controls in case-control studies and, if applicable, for exposed and unexposed groups in cohort and cross-sectional studies.

**Note:** An Explanation and Elaboration article discusses each checklist item and gives methodological background and published examples of transparent reporting. The STROBE checklist is best used in conjunction with this article (freely available on the Web sites of PLoS Medicine at http://www.plosmedicine.org/, Annals of Internal Medicine at http://www.annals.org/, and Epidemiology at http://www.epidem.com/). Information on the STROBE Initiative is available at www.strobe-statement.org.
